# High-resolution X-ray diffraction analysis of strain distribution in GaN nanowires on Si(111) substrate

**DOI:** 10.1186/s11671-015-0766-x

**Published:** 2015-02-06

**Authors:** Hryhorii Stanchu, Vasyl Kladko, Andrian V Kuchuk, Nadiia Safriuk, Alexander Belyaev, Aleksandra Wierzbicka, Marta Sobanska, Kamil Klosek, Zbigniew R Zytkiewicz

**Affiliations:** V. Lashkaryov Institute of Semiconductor Physics, National Academy of Sciences of Ukraine, Pr. Nauky 45, Kyiv, 03028 Ukraine; Institute for Nanoscience and Engineering, University of Arkansas, W. Dickson731, 72701 Fayetteville, AR USA; Institute of Physics, Polish Academy of Sciences, Al. Lotnikow 32/46, 02-668 Warsaw, Poland

**Keywords:** Nanowires, GaN, Deformation, X-ray diffraction profile, Kinematical theory

## Abstract

In this work, the influence of micro- and macro-deformation profiles in GaN nanowires (NWs) on the angular intensity distribution of X-ray diffraction are studied theoretically. The calculations are performed by using kinematical theory of X-ray diffraction and assuming the deformation decays exponentially from the NW/substrate interface. Theoretical modeling of X-ray scattering from NWs with different deformation profiles are carried out. We show that the shape of the (002) 2θ/ω X-ray diffraction profile (XDP) is defined by initial deformation at the NW's bottom and its relaxation depth given by the decay depth of the exponential deformation profile. Also, we demonstrate that macro-deformation leads to XDP shift, whereas micro-deformations are the cause of XDP's asymmetry and its symmetrical broadening. A good correlation between calculated and experimental XDP from self-assembled GaN NWs on Si(111) substrate was achieved by taking into account all parameters of micro- and macro-deformation profiles.

## Background

The wide range of unique properties of semiconductor GaN nanowires (NWs), along with various techniques of NW's growth, makes them promising candidates for nano-sized optoelectronics [1]. In recent years, a great deal of effort has been devoted to explore their optical, electrical, and structural properties. The main sources of deterioration of NW's properties are their crystalline imperfection and residual strain. Generally, NWs are considered almost strain-free crystalline-objects without extended defects that propagate into their structure [2-5]. In comparison with thick planar epilayers, where the mechanism of lattice accommodation is preferably plastic and where the formation of misfit dislocation networks takes place, NWs are considered to be predominantly free of dislocations [6,7]. At the same time, the process of deformation relaxation in NWs is not completely studied.

Over recent years, there have been only a few significant reports devoted to this subject. In [2], the assessment of macro- and micro-deformation in GaN NWs grown on Si(111) was performed with X-ray diffraction, where the NWs were found to be free of deformations on the macro scale but not on the micro scale. In comparison to self-assembled GaN NWs [2], an exponential relaxation of the macro-strain along the NW height was observed for top-down fabricated GaN NWs grown on silicon and sapphire substrates [3,4]. An exponential decay of the mean-squared micro-strain along the self-assembled GaN NW was considered to describe the X-ray diffraction peaks in [8]. Moreover, theoretical analysis of the elastic energy relaxation in NWs of different geometries grown on lattice mismatched substrates was performed assuming an exponential decay of strain [9].

In this letter, we study the influence of macro- and micro-deformation along the GaN NW's growth axis on the X-ray diffraction peak profile. We show that macro-deformations lead to X-ray diffraction profile (XDP) shift, whereas micro-deformations are the cause of XDP's asymmetry and its symmetrical broadening. This allows distinguishing the influence of macro- and micro-deformation components on XDP and thereby to extract them separately.

## Methods

Self-induced GaN NWs were grown by plasma-assisted molecular beam epitaxy (MBE) on a Si(111) substrate at approximately 760°C under highly nitrogen-rich conditions. Before the growth started, the substrate was exposed to a nitrogen flux for 30 min at the nitridation temperature of approximately 150°C. The procedure of plasma-assisted MBE growth of GaN NWs on Si(111) is described in [10,11]. The high-resolution X-ray diffraction measurements were performed by using PANalytical X'Pert Pro MRD diffractometer (PANalytical, Almelo, the Netherlands) equipped with a 1.6 kW X-ray tube (vertical line focus) with Cu*K*α_1_ radiation (*λ* = 1.540598 Å), a symmetric 4 × Ge(220) monochromator and a channel-cut Ge(220) analyzer.

## Results and discussion

**Figure 1:**
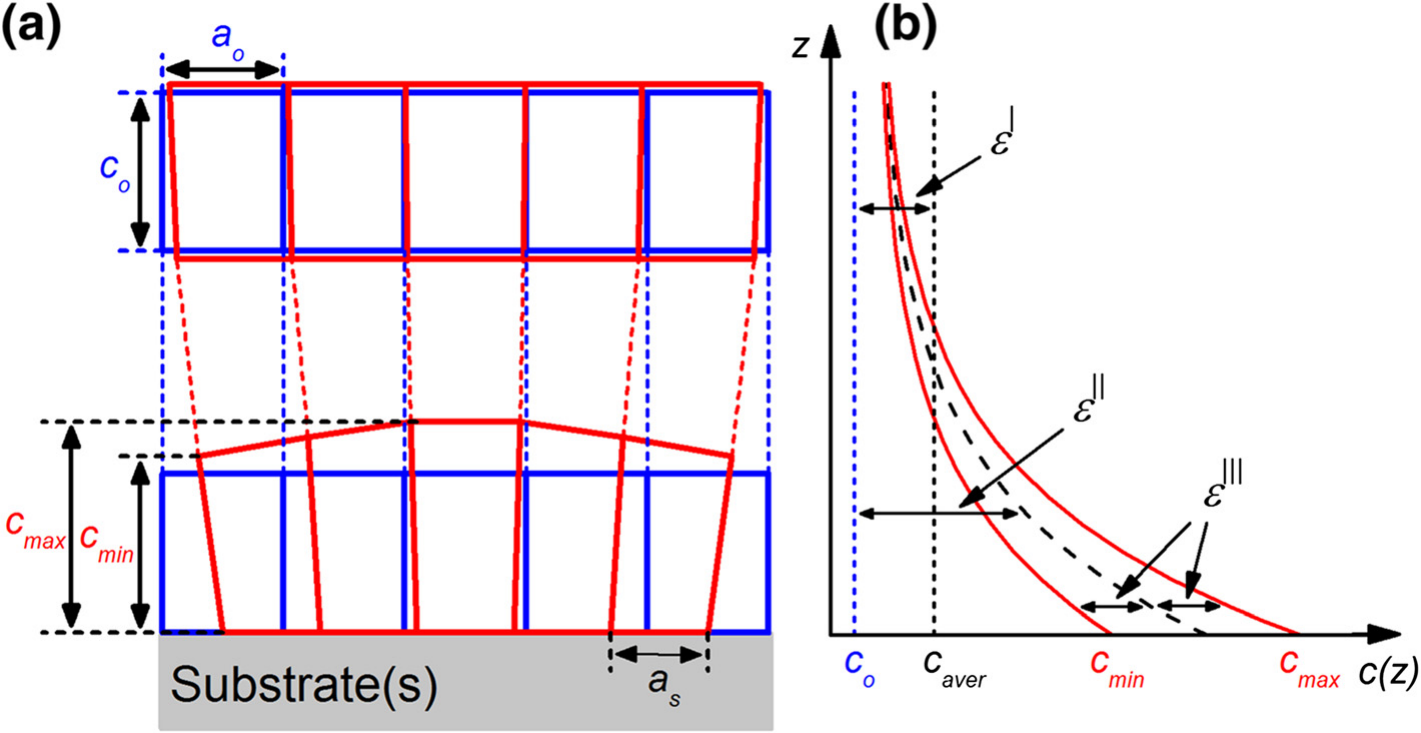
**Schematic illustration of the NW's deformation state.** Deformation at the top and NW/substrate interface **(a)** along with the out-of-plane lattice parameter as a function of NW's height **(b)**. Here, we show the case of tensile deformation along the NW's c-axis (red line) in comparison with non-deformed NW (blue line).

A large jump of deformation occurs at the GaN/substrate interface, due to the large mismatch of the lattice parameters and coefficients of thermal expansion between GaN and conventional substrates. As GaN NWs are bounded by free surfaces, the misfit deformation that is mainly concentrated at the NW/substrate heterointerface elastically relaxes to the NW's free sides. Therefore, the deformation of a whole NW and at the heterointerface will considerably differ, i.e., deformation decreases from the bottom to the top of NW, as schematically shown in Figure 1.

Depending on the crystal's considering volume, deformations are divided into two groups: macro-deformation (*ε*^|^) and micro-deformations (*ε*^||^ and *ε*^|||^). The macro-deformation *ε*^|^ is an integral deformation of a whole sample. On the other hand, deformations *ε*^||^ and *ε*^|||^ occur in a limited part of a crystal and lattice unit volume, respectively. We consider that NWs are mostly deformed at their bottoms, thus they can be assumed to be free of *ε*^|^. As it is shown in Figure 1b, for a particular height of NW, we define *ε*^||^(*z*) as the average lattice cells deformation along the NW diameter (see Equation 1), while *ε*^|||^(*z*) is the deformation fluctuation around the *ε*^||^(z) (see Equation 2):

1 $$ {\varepsilon}^{\left|\right|}(z)=\frac{c_{max}(z)+{c}_{min}(z)}{2{c}_o}-1, $$

2 $$ {\varepsilon}^{|||}(z)=\frac{c_{max}(z)-{c}_o}{c_o}-{\varepsilon}^{\left|\right|}(z)={\varepsilon}^{\left|\right|}(z)-\frac{c_{min}(z)-{c}_o}{c_o}, $$

with *c*_*o*_ *=* 0.51851 nm - bulk GaN lattice parameter; *c*_min_(*z*) and *c*_max_(*z*) are the minimal and maximal deviation from *c*_*o*_ of the out-of-plane lattice parameter along the GaN nanowire's diameter at NW's height *z*.

The deformation profiles of *ε*^||^(z) and *ε*^|||^(*z*) along the NW's growth axis are defined assuming exponential deformation decay [3,4,8,9]:

3 $$ {\varepsilon}^{\left|\left|/\Big|\right|\right|}(z)={\varepsilon}_o^{\left|\left|/\Big|\right|\right|}{e}^{-\frac{z}{L_R}}, $$

where *L*_*R*_ is the deformation relaxation depth, at which the deformation relaxes to *1/e* of the initial values (${\varepsilon}_o^{\left|\right|}$ or ${\varepsilon}_o^{\left|\Big|\right|}$), and *z* is the NW's height.

A simple simulation of XDP was performed to get information about the deformation distribution in GaN NWs. Here, we simulated a symmetric (002) 2θ/ω diffraction profile using the kinematical theory of X-ray diffraction. The deviations of the out-of-plane lattice parameter from the bulk value along the NW's height appear as variations in the length of the scattering vector *q* at which the Bragg's condition is satisfied. According to the geometry conditions of the scattering process for symmetric reflections [12], the length of *q* = *k*_in_ − *k*_out_ (where *k*_in_ and *k*_out_ are the incident and scattered wave vectors, respectively) is related to the incidence angle *θ* by:

4 $$ q={q}_z=2\left|k\right| \sin \left(\theta \right). $$

Taking into account the Bragg's law 2*d*_(00*l*)_ sin(*θ*) = *nλ*, we can present the length of the scattering vector proportional to the lattice parameter *c*(*z*):

5 $$ {q}^{\left(h=k=0\right)}=\frac{nl}{c(z)}, $$

where (*hkl*) are Miller indices and *n* is the diffraction order.

Thus, from Equations 4 and 5, one can see that the change in the lattice parameter *c*(*z*) along the NW height is the origin of intensity distribution in Equation 6, where the intensity was obtained by summing amplitudes reflected by each crystal plane:

6 $$ I(q)={\left|{\displaystyle \sum_i^N{F}_i \exp \left(-2\pi iq\left({z}_i+u(z)\right)\right)}\right|}^2, $$

where *F*_*i*_ is the structure factor of a plane at depth *z*_*i*_; *N* is the number of crystal planes and *u*(*z*) = 〈*u*(*z*)〉 ± *δu*(*z*). Here, 〈*u*(*z*)〉 = *ε*^||^(*z*) ⋅ *c*_*o*_ and *δu*(*z*) = *ε*^|||^(*z*) ⋅ *c*_*o*_ are the deviations of NW's lattice parameter *c*(*z*) from the bulk value *c*_o_, caused by deformations *ε*^||^(*z*) and *ε*^|||^(*z*), respectively.

**Figure 2:**
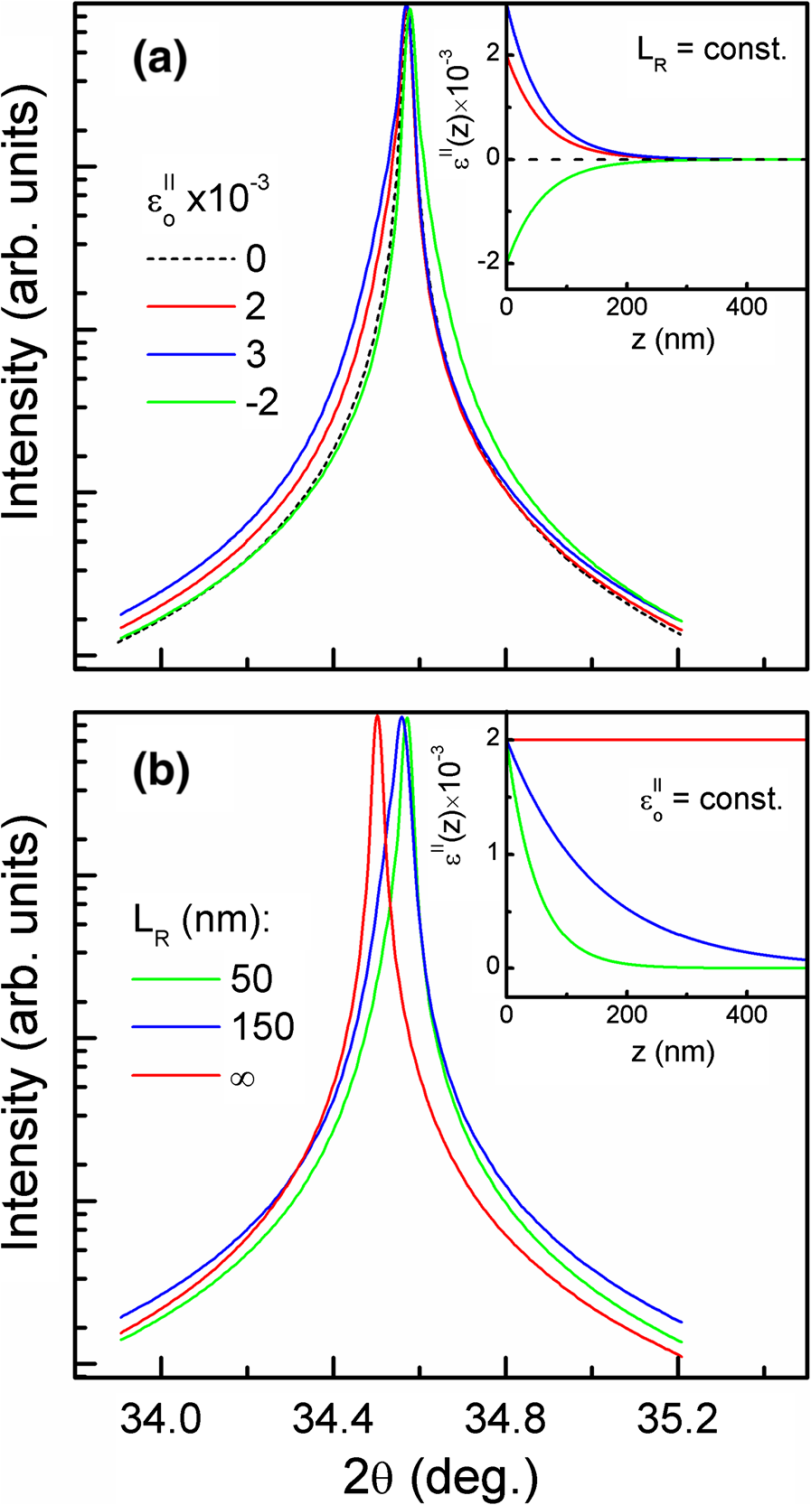
**The calculated (002) 2θ/ω XDPs for GaN NWs. (a)** The influence of the deformation magnitude (${\varepsilon}_o^{\left|\right|}$) at the NW/substrate interface. **(b)** The influence of the deformation relaxation depth (*L*_*R*_). The insets show the deformation profiles (defined by Equation 3) used for the calculation. The dashed line in **(a)** shows the XDP determined only by the size effect (without deformation).

Different types of deformation lead to specific changes in the XDP. In Figure 2, we demonstrate a calculated (002) 2θ/ω XDP for a 500-nm-long GaN NWs array affected only by micro-deformation *ε*^||^(*z*). We show how the magnitude of initial deformation ${\varepsilon}_o^{\left|\right|}$ (caused by the lattice misfit at the NW/substrate interface) and the deformation relaxation depth *L*_*R*_ (dependent on the aspect ratio of NW) contribute to the XDP. It is shown (Figure 2a) that for *L*_*R*_ = const, the increase of magnitude of micro-deformation at the NW/substrate interface $\left({\varepsilon}_o^{\left|\right|}\right)$ leads to asymmetrical XDP broadening, whereas the deformation sign (tensile or compressive deformation) defines the broadening direction. Similarly (Figure 2b), for ${\varepsilon}_o^{\left|\right|}=\mathrm{const},$ we demonstrate that the magnitude of deformation relaxation depth (*L*_*R*_) is another source of asymmetrical XDP broadening. Moreover, when *L*_*R*_ exceed some critical value (for a specific aspect ratio of NW), which leads to the XDP shift, we can conclude on the presence of macro-deformation *ε*^|^. Thus, the value of this deformation can be estimated directly from the magnitude of the XDP shift. It should be noted that the asymmetric broadening and shift of XDP for GaN NWs was observed experimentally in [3,4].

**Figure 3:**
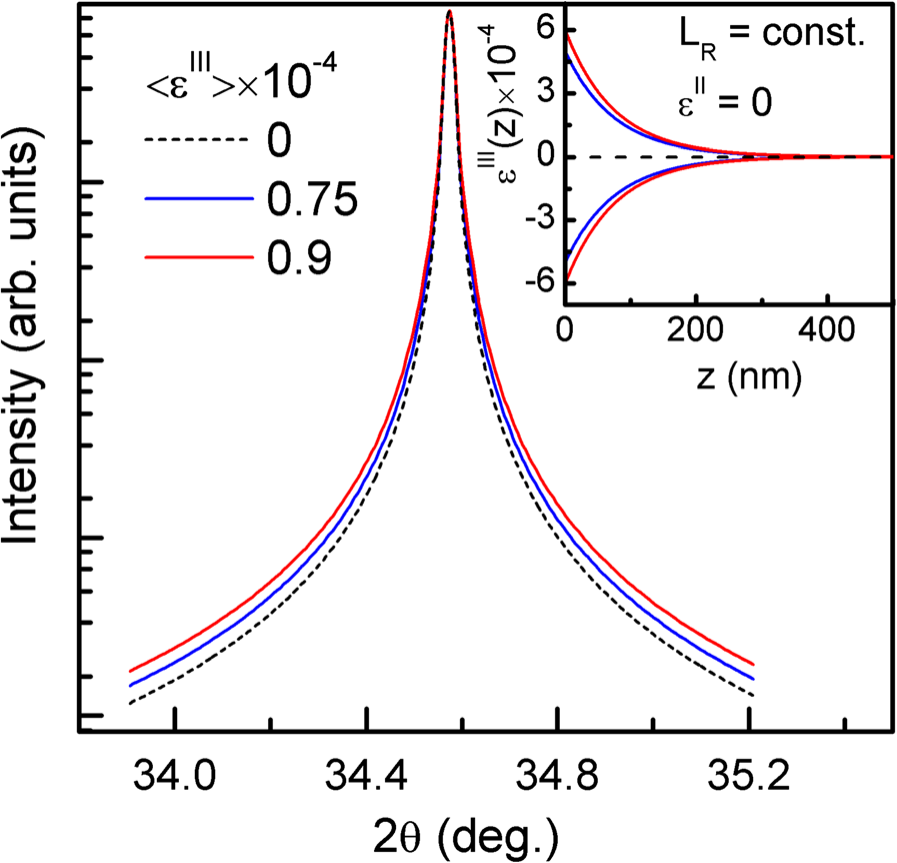
**The calculated (002) 2θ/ω XDPs for GaN NWs affected by the micro-deformation** ***ε*** ^***III***^ **(** ***z*** **)** **.** The deformation distribution profiles of *ε* ^*III*^(*z*) used for XDP calculation were defined by Equation 3 and are shown in the inset.

Next, we analyze the influence of only micro-deformation *ε*^|||^(*z*) (caused by the elastic strain relaxation at the free NW's surface) on the XDP's shape. According to the definition, this unsigned deformation *ε*^|||^(*z*) is the deformation fluctuation around the *ε*^||^(z), and it is described by the same law (see Equation 3). Thus, for *L*_*R*_ = const and ${\varepsilon}_o^{\left|\right|}=0,$ we calculated (002) 2θ/ω XDP for a 500-nm-long GaN NWs array affected only by micro-deformation *ε*^|||^(*z*). It is shown (Figure 3) that the increase of *ε*^|||^(*z*) magnitude leads only to symmetrical XDP broadening. It should be noted that coalescence of NWs and lattice distortion have a strong influence on deformation profile of *ε*^|||^(*z*) [8,13]. Actually, all kinds of deformations are not independent, and a nonlinear change of *ε*^|||^(*z*) deformation leads to an appearance of *ε*^||^(*z*) deformation. Moreover, the increase of micro-deformation (*ε*^||^(*z*)) relaxation depth *L*_*R*_ leads to the appearance of a macro-deformation (*ε*^|^). Thus, to fit the theoretical XDP from GaN NWs to the experimental one, the distribution of micro- and macro-deformation profiles should be taken into account.

**Figure 4:**
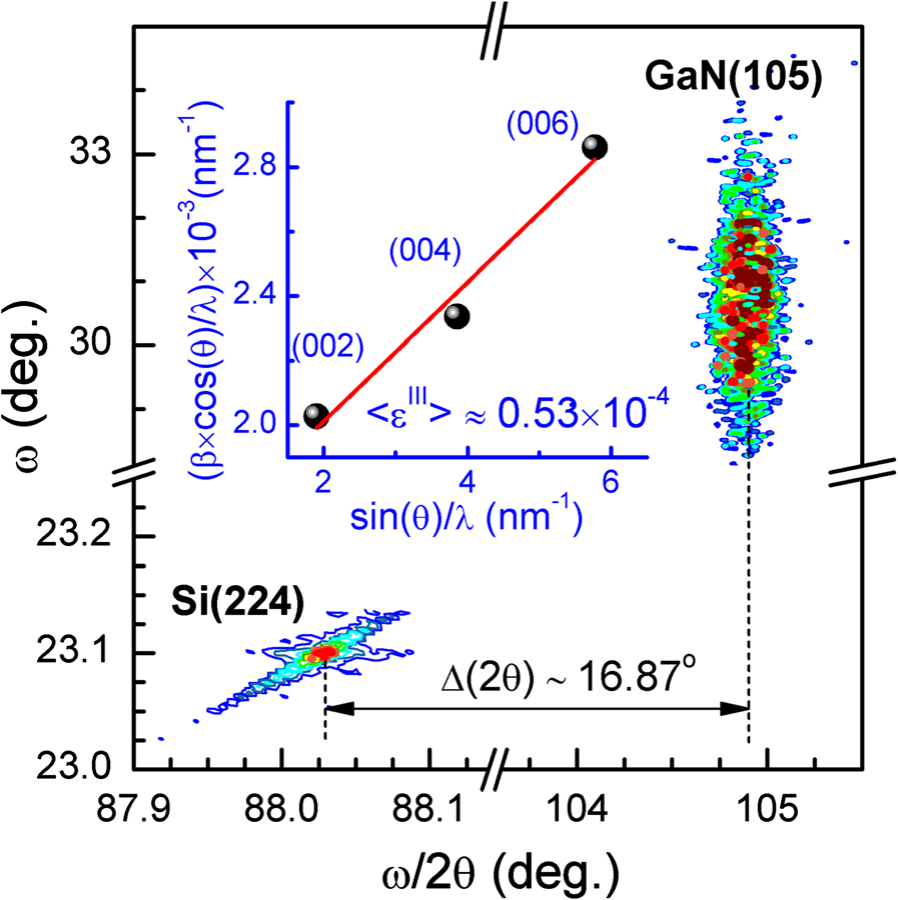
**The asymmetrical reciprocal space maps near the Si(224) and GaN(105) reflections.** The inset presents the Williamson-Hall plot for the symmetrical (*00 l*) GaN reflections (*l* = 2,4,6).

Then, we consider the simulation of XDP from self-induced GaN NWs grown on Si(111) substrate. In order to evaluate the macro- and micro-deformation state of GaN NWs, we first used a standard approach based on measurements of reciprocal space map (RSM) and a series of symmetrical (*00 l*) 2θ/ω reflections (*l =* 2,4,6). From the angular position of GaN(105) and Si(224) peaks on RSM (Figure 4), we conclude on almost fully relaxed state of GaN NWs on the macro scale (R = (Δ*θ*_exp_/Δ*θ*_bulk_) ⋅ 100 % ≈ 99 %). Here, Δ*θ*_exp_ and Δ*θ*_bulk_ are the differences between angular positions of GaN(105) and Si(224) peaks measured experimentally and their bulk values, respectively. On the other hand, the heterogeneous micro-deformation along the NWs c-axis and a small vertical correlation length (VCL) contribute to the broadening of the symmetrical 2θ/ω reflections in radial scan direction. These two effects have different dependencies on the diffraction vector and can be separated using Williamson-Hall (WH) plots [14,15]. The WH plot analysis (inset in Figure 4) gives the value of VCL equal to approximately 285 nm and the average micro-deformation approximately 0.53 × 10^−4^.

**Figure 5:**
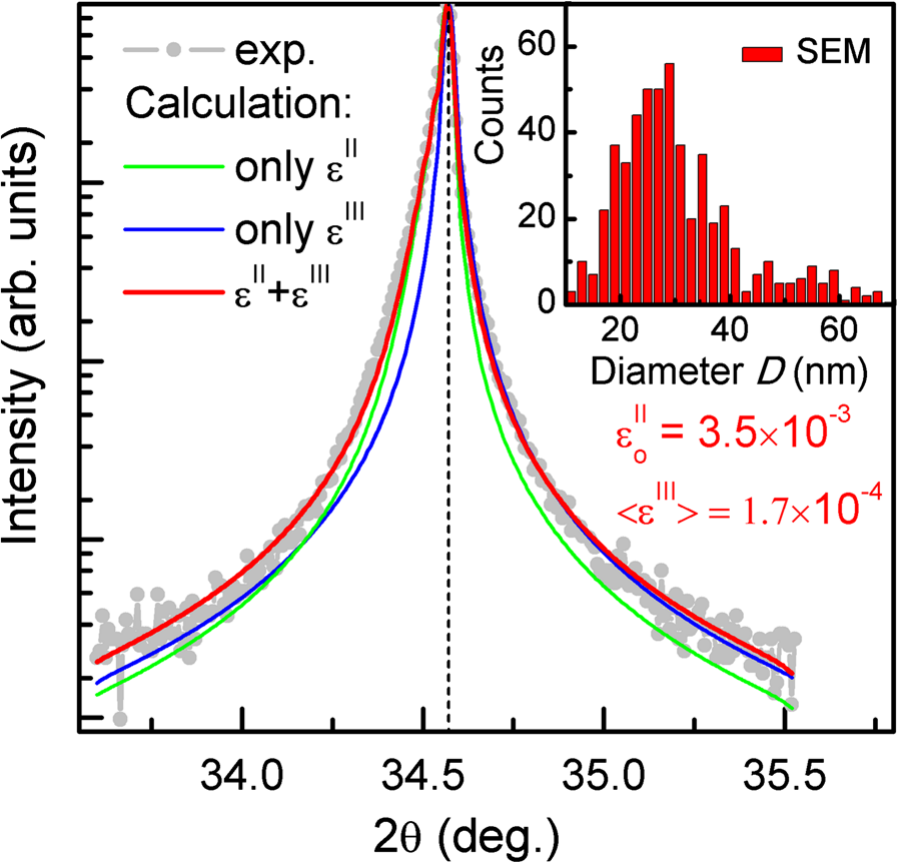
**Experimental and calculated 2θ/ω XDPs of the (002) reflection for GaN NWs.** The black dashed line indicates the peak position of bulk GaN. The inset NW's diameter distribution was estimated using top-view image of scanning electron microscopy (SEM).

Further, we calculated the XDP for a whole GaN nanowire ensemble taking into account the NW diameter distribution. We used the approach presented in [8], where the XDP of each NW is multiplied by *D*^*2*^ (where *D* is the NW's diameter) and the frequency of the corresponding nanowire diameter. Experimental and calculated (002) 2θ/ω XDPs of GaN NWs are shown in Figure 5. The simulated XDP (red line) is plotted along with the XDPs where either the *ε*^|||^(*z*) or *ε*^||^(*z*) is taken into account individually (blue and green line, respectively). It is demonstrated that in order to achieve a good fitting of experimental 2θ/ω XDPs, both components of micro-deformation should be considered. Moreover, since no peak shift is observed, we conclude that the NWs are free of macro-deformation *ε*^|^, which well correlates with results obtained from RSM. The large asymmetry of the red XDP indicates significant *ε*^||^(*z*) tensile micro-deformation at the bottom of the GaN NWs. The calculation provides the value of deformation relaxation depth *L*_*R*_ to be 1.4 times larger than the NW's diameter and the magnitude of *ε*^||^(*z*) of the order of 10^−3^, which are close to values obtained in [3,4,8]. Also, the fitting procedure gives the average NW's height *z* equal to 400 nm, which correlates well with values obtained from cross-sectional SEM image (not shown here). The average value of micro-deformation < *ε*^|||^(*z*) > ~ 1.7 × 10^− 4^ is in good agreement with the values presented in [2,13] for GaN NWs grown also on Si(111). Thus, in comparison with standard WH analysis based on FWHM of 2θ/ω-scans (which gives the average value of VCL and micro-strain fluctuation), the calculation of the full XDP allows to evaluate the macro- and micro-deformation depth profiles and more accurate value of the NW's height.

## Conclusions

In this work, the theoretical analysis of micro- and macro-deformation parameters that influence symmetrical (002) 2θ/ω XDP from GaN NWs is presented. It is demonstrated that the macro-deformation *ε*^|^ of a whole NW leads to the angular position shift of XDP. Inhomogeneous micro-deformation *ε*^||^(*z*) along the NW height leads to asymmetry of the XDP. The micro-deformation *ε*^|||^(*z*) fluctuation around the *ε*^||^(*z*) causes only broadening of XDP. Thus, our theoretical approach of 2θ/ω XDP calculation can be used for quantitative analysis of GaN NWs with different shapes and deformation states.
